# Red Beetroot Skin Powder Addition as a Multifunctional Ingredient in Nougat

**DOI:** 10.3390/antiox14060676

**Published:** 2025-06-01

**Authors:** Oana Emilia Constantin, Silvia Lazăr (Mistrianu), Florina Stoica, Roxana Nicoleta Rațu, Doina Georgeta Andronoiu, Nicoleta Stănciuc, Marija Banožić, Nada Ćujić Nikolić, Zorana Mutavski, Gabriela Râpeanu

**Affiliations:** 1 Integrated Center for Research, Expertise and Technological Transfer in Food Industry, Faculty of Food Science and Engineering, Dunarea de Jos University of Galati, 111 Domnească Street, 800201 Galati, Romania; econstantin@ugal.ro (O.E.C.); silvia.lazar@ugal.ro (S.L.); roxana.ratu@uaiasi.ro (R.N.R.); georgeta.andronoiu@ugal.ro (D.G.A.); nicoleta.stanciuc@ugal.ro (N.S.); 2 Department of Pedotechnics, Faculty of Agriculture, “Ion Ionescu de La Brad” Iasi University of Life Sciences, 3 Mihail Sadoveanu Alley, 700489 Iasi, Romania; florina.stoica@uaiasi.ro; 3 Department of Food Technologies, Faculty of Agriculture, “Ion Ionescu de La Brad” Iasi University of Life Sciences, 3 Mihail Sadoveanu Alley, 700489 Iasi, Romania; 4 Faculty of Agriculture and Food Technology, University of Mostar, Biskupa Čule bb, 88000 Mostar, Bosnia and Herzegovina; marija.banozic@aptf.sum.ba; 5 Institute for Medicinal Plants Research “Dr. Josif Pančić”, Tadeuša Košćuška 1, 11000 Belgrade, Serbia; ncujic@mocbilja.rs (N.Ć.N.); zmutavski@mocbilja.rs (Z.M.)

**Keywords:** *Beta vulgaris* L., red beet skins, antioxidants, betalains, nougat

## Abstract

Beetroot (*Beta vulgaris* L.) is a plant grown for its roots, which are used to obtain sugar, feed animals, and for human use. Beetroot skin, a by-product of food processing, is a significant source of bioactive compounds, including dietary fiber and antioxidants. The primary objective of this work was to utilize beetroot skin powder to produce value-added nougat. Analytical methods, like antioxidant activity tests, proximate analysis, and sensory assessments, are used to determine the impact of beetroot skin powder on the final product. The beetroot skin powder extract had a remarkable content of phytochemicals and antioxidant activity. The inhibitory effect of the extract was tested on enzymes linked to metabolic syndrome, oxidative stress, and inflammation. The beetroot skin powder extract inhibited α-glucosidase, α-amylase, lipase, and lipoxygenase enzymes. The characterization of value-added nougat illustrates the multifunctionality of beetroot peel powder within its composition, serving as a significant source of natural compounds with antioxidant, coloring, and flavoring properties. This enhances sensory attributes, including color, aroma, and texture, augmenting product diversity and consumer appeal. This is evidenced by the increase in the total content of betalains (3.77 ± 0.09 mg/g DW.) and polyphenols (69.48 ± 2.88 mg GAE/100 g DW.), which lead to high antioxidant activity (73.89 ± 3.65 mM Trolox/100 g DW.) for the nougat sample with 6% added beetroot powder. Thus, beetroot skin powder replaced chemically synthesized additives with antioxidants and natural pigments, improving life quality and implicitly capitalizing on beetroot processing by-products, supporting circular economy principles at the global level.

## 1. Introduction

The minimally processed food sector generates substantial plant food waste daily, resulting in significant economic and nutritional losses and a substantial environmental impact. This waste material is becoming increasingly recognized as a natural reservoir of bioactive substances, which can be utilized to extract elements with both significant nutritional and commercial value, such as fibers and pigments. Within this particular framework, scientists have directed their attention toward using this waste to produce novel food items and ingredients [1].

Beetroot (*Beta vulgaris* L.), an Amaranthaceae plant, is one of the most antioxidant-rich due to its betalains and phenolic content [2]. Beetroot is rich in folate, which protects against congenital malformation; iron, which prevents and treats anemia; and dietary fiber, which improves colon health [3].

Betalains are water-soluble pigments approved by the European Union for various applications in the food industry, coded as E-162 [4,5,6]. Betalains are a family of pigments generated from betalamic acid, including red–violet betacyanin and yellow–orange betaxanthin, both water-soluble and nitrogen-containing. Betanin, the primary betacyanin, makes up to 90% of the red color in beetroot [7]. According to recent studies, these pigments inhibit food lipid oxidation and have numerous benefits for the body, such as cardioprotective, hepatoprotective, anti-inflammatory, antiproliferative, and antimicrobial effects [8,9].

Due to these health advantages, the food industry views betalains as natural nutritional supplements, colorants, or additives. For instance, betalains have been used in a variety of foods that are mostly made from red beetroot, which has been used commercially, such as yogurt, ice cream, and cakes [10,11,12].

The processing of red beetroots results in an important amount of vegetable by-products. These important sources of flavor compounds, dyes, and natural antioxidants can replace chemically synthesized additives in the composition of food products, increasing the quality of life and ensuring the circular economy worldwide. Red beetroot, noted for its vivid hue and health-enhancing phytonutrients, produces considerable skin waste during processing, especially in juice and puree manufacturing. Beetroot skins are abundant in dietary fiber, betalains (specifically betacyanins and betaxanthins), polyphenols, and other micronutrients, which exhibit antioxidant, anti-inflammatory, and antidiabetic effects [13]. Beetroot by-products are rich in betalains and phytochemicals, making them suitable for various food applications. These by-products are an abundant and cost-effective source of dietary fiber [14]. Utilizing beetroot by-products as inexpensive food constituents can minimize the amount of food waste. Thus, plant residues have the potential to serve as a nutraceutical resource for the development of functional foods [15]. Therefore, adding beetroot by-products to food products like nougat could be a cost-effective approach.

Nougat, a widely liked confectionery product composed of sugar, egg whites, and almonds, is conventionally valued for its texture and flavor rather than its nutritional advantages [16]. Nonetheless, consumer demand for functional and clean-label confections has prompted initiatives to enhance their nutritional profile without compromising sensory appeal. Integrating red beetroot skin powder into nougat formulations may provide an effective method to incorporate natural colorants, dietary fiber, and antioxidants, thus enhancing the functional qualities of this confectionery and elevating it to a value-added product.

In addition, using bioactive compounds from beetroot skins as flavoring substances, colorings, and natural antioxidants could provide antioxidant protection to food products and improve sensory characteristics, thus increasing the attractiveness and diversity of food products among consumers [17].

For many consumers, nougat is a dessert reminiscent of childhood, characterized by a sweet taste and different textures, a sugary food product obtained from sugar, glucose syrup, honey, starch, or egg white, with or without additions of nuts, almonds, candied fruits, raisins, cocoa, flavors, etc. To increase diversity, the classic recipes for obtaining the nougat are based on different ingredients but also with additions of chemically synthesized additives (thickening agents, loosening agents, preservatives, taste enhancers, flavors, and dyes) that might exert a cumulative impact on the human system and can lead to adverse health consequences [18].

In this context, beetroot waste might be a viable substitute for other valuable components, such as plant-based by-products high in bioactives and naturally colored. This study is based on obtaining a new type of nougat by incorporating the powder from the beetroot skins (*Beta vulgaris* L.) as a source of bioactive components and natural antioxidants. Furthermore, the effects of integrating beetroot skin powder on the phytochemical content, sensory characteristics, color, and textural properties of the nougat samples were also studied. Nougat, made with red beetroot skin powder, is a unique and sustainable functional confectionery. Beetroot skin, generally discarded as a food processing by-product, increases nougat’s nutritional, sensory, and shelf-life characteristics with its dietary fiber, natural colors (betalains), and antioxidants. It enhances color, texture, antioxidant capacity, clean label trends, and recycled ingredient use. This innovation meets consumer demand for healthy, ecologically friendly food products, promising value-added confectionery manufacture.

## 2. Materials and Methods

### 2.1. Materials and Reagents

All ingredients used for the nougat were purchased at a local market, including honey, sugar, egg whites, lemon juice, and salt. Ethanol, methanol, glacial acetic acid, HPLC purity, 2,2-diphenyl-1-picrylhydrazyl (DPPH), 6-hydroxy-2,5,7,8-tetramethylchromane-2-carboxylic acid (Trolox), Folin–Ciocalteu reagent, sodium hydroxide, sodium carbonate, and gallic acid; α-amylase from *Aspergillus oryzae*; α-glucosidase from *Saccharomyces cerevisiae* (EC 3.2.1.20); soybean lipoxidase (*Glycine max*) type I-B, ≥50,000 units/mg protein; ρ-nitrophenyl-α-D-glucopyranoside; pancreatic lipase (111.5 units/mg protein); starch; 3,5-dinitrosalicylic acid (DNS); sodium phosphate buffer solution (PBS); quercetin ≥ 95% (HPLC); orlistat ≥ 98%; and acarbose (purity ≥ 95%) were obtained from Sigma Aldrich (Steinheim, Germany). All the reagents used in the experiments were of analytical grade.

### 2.2. Beetroot Peel (BP) Powder Obtaining and Its Chemical Composition

The red beets were purchased from a local producer in the geographical area of SE Romania. The plant roots were washed, and the skins were removed in a thin layer with a scalpel. Next, the beetroot skin was washed with ultrapure water, blotted dry with a tissue, and freeze-dried (Christ Alpha 1-4 LD plus, Osterode am Harz, Germany). Finally, the dried skins with a relative humidity of 10% were ground using an MC 12 grinder (Stephan, Germany). The next step was to package the powder using polymer film and keep it at 20 °C until the characterization study.

The AOAC methodology was used to measure the beetroot skin powder’s moisture, ash, fat, protein, fiber, and carbohydrate (by difference) [19]. Pomeranz et al.’s [20] oven-drying method at 105 °C was used to measure moisture. A furnace at 600 °C heated a pre-weighed crucible with 5 g of sample for 4 h to measure the ash content. Weighing the desiccated item determined the ash content [21]. The gravimetric Soxhlet method quantified the fat content [19]. The Kjeldahl method was employed to ascertain the protein content, which involved multiplying the nitrogen (N) content by 5.7 [22]. Utilizing an enzymatic–gravimetric methodology described by Chantaro et al. [23], the crude fiber content was quantified. The total carbohydrate content was calculated by difference: [100 − (moisture + protein + fat + ash) %].

### 2.3. Color Analysis

Starting with a white calibration plate, a MINOLTA Chroma Meter CR-410 (Konica Minolta, Osaka, Japan) evaluated the beetroot skin powder’s color. The color data are represented using the CIE L*, a*, and b* values, where L* indicates lightness, with a ranging from 0 (black) to 100 (white); a* ranging from −a* (greenness) to +a* (redness); and b* spanning from −b* (blueness) to +b* (yellowness).

### 2.4. Mineral Analysis

One gram of beetroot skin powder was subjected to digestion with nitric acid (HNO_3_) and hydrochloric acid (HCl) in an 8:2 ratio at 180 °C for 40 min. The material was dissolved in a standard flask in 25 mL of deionized water. The concentrations of minerals Ca, Na, P, Mg, K, Fe, Cu, Zn, and Mn were quantified using atomic absorption spectrophotometry [24].

### 2.5. Extraction of Beet Skin Powder

A total of 1 g of powdered red beet peel, 9 mL of 60% (*v*/*v*) ethanol, and 1% citric acid were used in the extraction process, which had an acid–solvent ratio of 1:8. In a sonication water bath (Smart Sonic MRC Scientific 193 Instruments, Holon, Israel), extractions were carried out for 50 min at 35 °C. The extracts were centrifuged at 6000 rpm for 10 min at 4 °C using a Hettich Universal 320R (Germany). The resultant supernatant was subsequently utilized for further characterization.

#### 2.5.1. Determination of Total Betalain Content

Stintzing et al.’s [25] spectrophotometric approach with small modifications was used to measure the betalain amount in the beetroot skin powder extract using a UV-Vis Biochrom Libra S22 (UK) spectrophotometer. The absorbances were measured at 538 nm and 480 nm for betacyanins and betaxanthins, respectively. The betalain concentration was calculated based on Equation (1):

(1) $$\mathrm{Betalains}\;\left(\mathrm{mg}/g\right)=\frac{A\;\times \;\mathrm{MW}\;\times \;\mathrm{DF}\;\times \;\mathrm{Vd}}{e\;\times \;L\;\times \;\mathrm{Wd}}$$

where A is the absorbance of the sample (A at 538 nm for betacyanins and A at 480 nm for betaxanthins), DF is the dilution factor, L is the cuvette path length (1 cm), Wd (g) is the beetroot skin powder quantity, and Vd is the beetroot skin powder solution volume. Betacyanins and betaxanthins were quantified using their molecular weight (MW) and molar extinction coefficients (e): MW = 550 g/mol; e = 60,000 L/mol cm in H_2_O for betacyanin and MW = 308 g/mol; e = 48,000 L/mol cm in H_2_O for betaxanthins.

#### 2.5.2. Determination of Total Phenolic Content

The total phenolic content was assessed spectrophotometrically using the Folin–Ciocalteu method, according to Constantin et al. [26]. To summarize, 200 µL of the extract, 15.8 mL of deionized water, and 1 mL of Folin–Ciocalteau reagent were thoroughly combined. The mixture received 3 mL of 20% sodium carbonate after 10 min. Following a 60 min incubation at ambient temperature and in darkness, the absorbance at 765 nm was ascribed to the blank. The total phenolic content in the sample was evaluated by measuring the extract’s absorbance against a gallic acid standard curve (y = 1.6991x − 0.0256, R^2^ = 0.9837). The total phenolic content was expressed as milligrams of gallic acid equivalents (GAE) per gram of dry weight (mg GAE/g dw).

#### 2.5.3. Determination of Antioxidant Activity

DPPH assay. According to Lazăr et al. [27], the antioxidant activity was determined using DPPH free radical scavenging activity. Beetroot skin extracts proved their free radical scavenging potential by bleaching the stable DPPH radical. With 3.9 mL of 0.1 M DPPH in methanol, the blank’s absorbance was 515 nm. Add 3.9 mL of 0.1 M DPPH solution and 100 µL of extract to the reaction mixture. After 30 min at room temperature in the dark, the mixture’s 515 nm absorbance was measured. The calibration curve reference was Trolox. The antioxidant activity was expressed as µmoles of Trolox equivalents per gram of dry weight (µM of Trolox/g dw).

ABTS assay. The Roman et al. [28] technique was used to measure ABTS radical cation scavenging. A total of 5 mL of the 7 mM ABTS and 2.45 mM K_2_S_2_O_8_ mixture was maintained overnight, after diluting with 50% ethanol in order to obtain the solution absorbance of 0.7 at λ = 734 nm. After mixing 1 mL of diluted ABTS with 10 µL of powder extract, the absorbance was measured at λ = 734 nm. Results are reported in mM Trolox/g DW using a calibration curve plotting % ABTS scavenged against Trolox concentration.

#### 2.5.4. HPLC Investigation of the Betalains from the Beetroot Skin Extract

To identify and quantify the betalain pigments in the beetroot skin extract, chromatographic analysis was carried out using the Thermo Finnigan Surveyor HPLC system, controlled by Xcalibur software version 2.0.7 (Finnigan Surveyor LC, Thermo Scientific, Waltham, MA, USA), according to Constantin et al. [26]. The mobile phases were solvent A (0.012% aqueous formic acid) and solvent B (0.012% and 5% acetonitrile). The flow rate was 1 mL/min for a 10 μL sample injection. A Synergy 4u Fusion-RP 80A column (150 × 4.6 mm, 4 μm) conducted the separation at 30 °C. Chromatograms were obtained at 538 nm; retention times and comparisons to the standard were undertaken to identify the betanin.

#### 2.5.5. In Vitro Enzyme Activity Inhibition of the Beetroot Skin Extract

The beetroot skin powder extract was tested on enzymes such as α-glucosidase, α-amylase, lipase, and lipoxygenase. Spectrophotometric analysis was performed using the modified Stoica et al. [29] procedure to determine the beetroot skin extract’s in vitro inhibitory activity on the four enzymes under test. To a volume of 50 μL of extract (0.5, 1, and 5 μg/mL extract diluted) we added 50 μL of α-amylase solution (1 mg/mL in 0.1 M PBS, pH = 6.9)/α-glucosidase (1 mg/mL in 0.1 M PBS, pH = 6.9)/pancreatic lipase (1 mg/mL in 0.1 M PBS, pH = 8.0)/pancreatic lipase (1 mg/mL in 0.1 M PBS, pH = 8.0). The samples were stored for 5 min at room temperature. After that, the following were added to each sample depending on the enzyme used: 100 μL 1% concentration starch solution (α-amylase), 50 μL ρ-nitrophenyl-α-D-glucopyranoside (α-glucosidase), 0.01 M p-nitrophenyl palmitate, with Triton X-100 and Arabic gum (pancreatic lipase), and 50 μL 0.05 mM linoleic acid dissolved in 0.1 M PBS (pH = 9.0) (lipoxygenase). Subsequently, the samples were incubated for another 20 min at 37 °C. After incubating for α-amylase, 200 μL of dinitrosalicylic acid (DNS) reagent was added to the reaction mixture, then the samples were heated to 100 °C for 5 min in a water bath. Before the samples were read, they were diluted with 2 mL of distilled water. In the case of α-glucosidase, 800 μL of 0.2 M sodium carbonate was added to the reaction mixture after incubation. For pancreatic lipase, the samples were diluted with 1 mL of 0.1 M PBS at pH = 8.0, and for lipoxygenase, the samples were diluted with 2 mL of 0.1 M PBS at pH = 9.0. The absorbances were then read with a double-beam UV-VIS spectrophotometer (Libra S22 Biochrom, United Kingdom) at λ = 540 nm (α-amylase), λ = 405 nm (α-glucosidase), λ = 400 nm (pancreatic lipase), and λ = 234 nm (lipoxygenase). The extract’s inhibitory effect was quantified using the IC50 value (µg/mL), representing the concentration at which the extract inhibits 50% of the enzyme activity. The standard inhibitors used as references were acarbose, orlistat, and quercetin.

### 2.6. Obtaining the Nougat Supplemented with Beetroot Skin Powder

Ingredients % (*w*/*w*) for added-value nougat were: sugar (52%), honey (26%), egg white (16%), lemon juice (2%), salt (0.2%), and beetroot skin powder previously hydrated with water in a 1:1 ratio (H1—2%, H2—4%, and H3—6%), water (the rest, up to 100%).

The following steps were followed to create the nougat with additional value: initially, the egg whites were mixed with the sugar at a high speed for 12–15 min until the composition became foamy and the sugar melted. Then, the lemon juice was mixed for another 5 min until a dense, glossy, firm foam was obtained. The foam obtained was transferred to a bain-marie vessel, and liquid honey was added in a thin thread, homogenizing for 60 min until the foam lost its volume and the consistency became sticky. Then, the product was removed from the bain-marie and tempered for 10 min. After this was added, the powder from the beetroot skins (relative to the quantity of the product) was hydrated afterward so that the color and texture were consistent. The composition was then poured between two sheets of wafers and kept under refrigerated conditions (4–5 °C) for analysis. A control sample was likewise prepared using the same methodology but without adding beetroot skin powder.

#### 2.6.1. Phytochemical and Physicochemical Characterization, and the Evaluation of the Antioxidant Potential of Supplemented Nougat

AOAC techniques assessed nougat samples’ moisture, protein, lipid, insoluble fiber, ash, carbohydrate contents, and energy value [19]. The betalains, phenolic content, and antioxidant activity of nougat samples (control sample and supplemented with beetroot skin powder) were evaluated using the previously mentioned methods.

#### 2.6.2. Storage Stability of Phytochemical Compounds

The nougat samples were preserved in light-resistant glass containers at ambient temperature protected from light exposure. Over a 21-day storage period, they were analyzed for their bioactive content (betalains and polyphenols) and DPPH scavenging activity, following the methods previously outlined.

#### 2.6.3. Determination of the CIELAB Colorimetric Parameters

The nougat formulations enriched with beetroot skin powder and the control sample were analyzed for CIELAB colorimetric parameters using a portable colorimeter with illuminator C (Chroma Meter, model CR-410, Konica Minolta, Osaka, Japan). It was calibrated with a white reference tile prior to each measurement. The Chroma (Equation (2)), hue angle (Equation (3)), and total color difference (ΔE) (Equation (4)) were calculated using the formula provided by Nistor et al. [30].

(2) $$\mathrm{The}\;\mathrm{Chroma}\;=\sqrt{{\left(a^{*}\right)}^{2}+{\left(b^{*}\right)}^{2}}$$

Hue angle = arctan (b*/a*) for quadrant I (+a*, +b*) (3)

(4) $$\Delta E\;=\sqrt{{\left(L^{*}-L_{0}\right)}^{2}+{\left(a^{*}-a_{0}\right)}^{2}+{\left(b^{*}-b_{0}\right)}^{2}}.$$

#### 2.6.4. Analysis of the Textural Parameters of Nougat Samples

Using a CT3-1000 texture analyzer (Brookfield Ametek, Chandler, AZ, USA), the textural properties of enriched nougat samples were investigated using the texture profile analysis (TPA) technique. Double dispersion in a 38.1 mm acrylic cylinder sample yielded 20 mm depth. The trigger load was 0.067 N, the load cell was 9.8 N, and the test speed was 1 ms. The textural parameters of firmness, cohesiveness, elasticity, adhesiveness, and chewability were calculated using TexturePro CT software V1.5. Every sample underwent three determinations. Prior to testing, the samples were left at room temperature for two hours.

#### 2.6.5. Mineral Analysis of Nougat Samples

The mineral compositions of the samples were examined via atomic absorption spectrometry (ContrAA 700, Analytik Jena, Jena, Germany) with a flame atomizer device. The analysis used MiniWAVE Microwave (SCP Science, Baie-d’Urfé, Canada) digestion equipment [24]. The mineral elements analyzed in the enriched nougat samples comprised K, Ca, P, Mg, Fe, Mn, Cu, Zn, and Na. The findings are presented in mg/100 g of dry weight.

#### 2.6.6. Sensory Analysis of Nougat Samples

Ten untrained customers, consisting of 90% women and 10% men aged 25–60 years, conducted a sensory evaluation of supplemented nougat samples. The participants were informed about this study’s primary purpose and the protocols for managing personal data. The sensory evaluation test was performed, considering Decision No. 19 from 16.04.2025 of the Dunarea de Jos University Ethics Commission. The panelists received directions to assess the color, appearance, odor, flavor, taste, texture, aftertaste, and general acceptability of supplemented nougat samples using a 9-point hedonic scale, where 1 represents strong dislike and 9 represents strong liking.

Alongside water and crackers for palate cleansing and consumption between sample analyses, the samples were presented at ambient temperature. The average score each panelist provided for each attribute was used to analyze the data.

### 2.7. Statistical Analysis of Data

The data presented in this study are the mean of three independent analyses, followed by the standard deviation of the mean. Analysis of variance was used to find significant differences between the results (ANOVA). Minitab 18 software was used to apply Tukey’s test to identify the significant pairwise comparisons. Statistical significance was defined as *p*-values of *p* < 0.05 for all tests. This investigation used the XLSTAT program, version 2024.3, for Principal Component Analysis.

## 3. Results and Discussion

### 3.1. Phytochemicals Extraction and Characterization of Beetroot Skin Powder

The beetroot skin powder extract’s phytochemical composition and antioxidant capacity were ascertained. The content of bioactives obtained was 1.24 ± 0.04 mg/g dw of betalain and 212.14 ± 1.49 mg GAE/g dw of polyphenols. The extract also demonstrated a 33.42 ± 1.12 µM Trolox/g dw DPPH radical scavenging capability.

The results comply with the information published in other studies. According to Vulić et al. [15], beetroot pomace extract contained 0.75 to 3.75 mg/g dw betalains, depending on genotype. Aqueous beetroot skin extract has a total phenolic concentration of 399.6 μg GAE/mL extract and 218.3 mg GAE/g dw, according to the reports by Raikos et al. [31] and Jasna et al. [32]. According to Maqbool et al. [33], beetroot skin extract exhibited a range of radical scavenging activities, from 70.12 ± 0.90% to 91.62 ± 0.90%.

A lighter color is indicated by greater L* values, which were measured at 36.90 ± 0.43. Because of the larger amount of total betalains, the a* parameter shows a tilt toward red. A positive value indicates a trend toward yellow hues in the powder. The b* parameter is a measure of the blue-to-yellow intensity. All data points were plotted in the first quadrant (+a*, +b*), based on the results of the a* and b* values, revealing a high prevalence of yellow and red tones, typical of total betalains [34].

The chemical composition of beetroot skin powder is shown in Table 1. The moisture content was 8.05%, whereas ash, fat, carbohydrates, protein, and fiber contents were 6.51, 0.59, 67.74, 17.12, and 33.10% on a dry basis, respectively. Šeremet et al. [35] found similar results for beetroot skin powder; therefore, the protein, fat, dry matter, total sugar, and total fiber in beetroot skin were approximately 18.3, 0.60, 90.80, 12.50, and 33.60%, respectively, on a dry basis. Shuaibu et al. [36] found that the peels of beets have 4.1% protein and 6.98% fiber while noting a higher ash content of 10.6% in the beetroot skin compared with our study.

An atomic absorption spectrophotometer measured the mean mineral concentrations in beetroot skin powder, as shown in Table 1. The results indicate that the quantity of K yielded substantial results in beetroot skin powder, followed by P, Na, Ca, Mg, Fe, Mn, Zn, and Cu, exhibiting considerable amounts.

Beetroot skin is rich in minerals, including manganese, sodium, potassium, magnesium, iron, and copper [37]. Alshehry [38] noted that potassium (26.0 mg/g) is the primary component in beetroot powder, followed by sodium (6.26 mg/g) and phosphorus (3.50 mg/g). These discrepancies could be due to different geographic origins or genotypes under study, extraction methods, and analytical procedures [39]. However, various extraction circumstances (such as solvent type, temperature, pH, and light intensity) and testing techniques might affect the phytochemical content of beetroot skin extracts. Genetic and agronomic factors can also affect this phytochemical composition.

The research has shown that beetroot skin powder is a highly advantageous source of phytochemicals with antioxidant characteristics. These cheap, raw materials can be included in foods as a natural component to efficiently diminish the presence of agro-industrial residues.

### 3.2. HPLC Investigation of Beetroot Skin Powder

**Figure 1:**
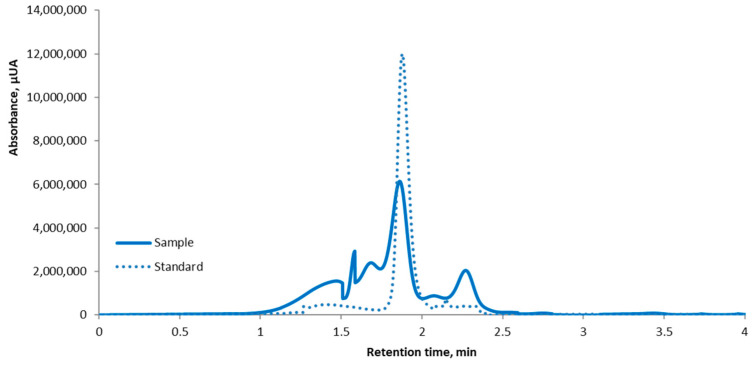
Quantification of betanin using HPLC chromatography in beetroot skin powder extract.

A preliminary High-Performance Liquid Chromatography (HPLC) analysis was also presented in our previous study [26], which showed that beetroot skin had a distinct peak at a retention time of 1.87 ± 0.2 min. Additionally, our findings (Figure 1) concur with those of earlier studies by Kujala et al. [40] and Rotich et al. [41]. According to the findings, betanin is the principal component that may be found in significant quantities in the peels and pomace of *B. vulgaris* L. The content of betanin in beetroot powder extract was 8.2 mg/g dw. The findings are supported by Prieto-Santiago et al. [42], who established that the total betanin content in red beet varied from 2.50 to 8.50 mg/g dw. Rotich et al. [41] detected an average of 3.81 ± 0.30 mg/g of betanin in *B. vulgaris* L. pomace from fruit juice sellers in Eldoret, Kenya, by the Soxhlet extraction method followed by HPLC-UV analysis.

### 3.3. In Vitro Enzyme Activity Inhibition

Beetroot skin powder extract showed a high potential to inhibit the tested enzymes at relatively low concentrations (0.5, 1, and 5 μg/mL) (Table 2). Beetroot skin powder extract showed inhibitory activity, indicated by IC50 values (μg/mL) of 4.22 ± 0.40 μg/mL for α-amylase, 3.24 ± 0.27 μg/mL for α-glucosidase, 1.05 ± 0.23 μg/mL for lipase, and 5.24 ± 0.59 μg/mL for lipoxygenase, compared to the standard inhibitors acarbose (IC50 2.69 ± 0.08; IC50 1.78 ± 0.06), orlistat (IC50 3.35 ± 0.24), and quercetin (IC50 2.40 ± 0.10).

The skin powder extract displayed activity against α-amylase and α-glucosidase, with IC50 values of 1.78 ± 0.06 and 2.69 ± 0.08 μg/mL, respectively. This suggests that by increasing the bioavailability of the bioactive compounds (flavonoids, betalains) in beetroot skin powder extract in the human body, they could be involved in reducing glucose metabolism since α-amylase is an enzyme that catalyzes the hydrolysis of starch into simple sugars [43].

In contrast to the standard acarbose (IC50 = 27.104 ± 0.270 μg/mL), flavonoid-rich extracts from *B. vulgaris* roots (IC50 = 73.062 ± 0.480 μg/mL) demonstrated moderate activity against α-amylase in additional studies. Compared to acarbose, these extracts showed stronger activity against α-glucosidase (IC50 = 17.389 ± 0.436 μg/mL) and exceeded it (IC50 = 37.564 ± 0.620 μg/mL) [44]. The *B. vulgaris* extracts showed inhibition effects (from 40.87 to 48.45% at 500 μg/mL) on pancreatic lipase [45]. Also, the lipoxygenase activity was inhibited by betalain red beetroot extract (IC50 = 1.33 mg/mL) [46]. Oboh et al. [47] determined the α-amylase and α-glucosidase inhibitory activity of freeze-dried beetroot juice. The percentage inhibition and IC50 value of beetroot juice revealed that it inhibits α-amylase (26% to 73%; IC50 = 1.78 ± 0.13 mg/mL) and α-glucosidase (53% to 85%; IC50 = ±0.73%; dose-dependent). Acarbose (control) inhibited α-amylase with an IC50 of 0.18 ± 0.02 mg/mL, while α-glucosidase had an IC50 of 0.22 ± 0.01 mg/mL.

These findings and the observed antioxidant activity in the beetroot skin powder extract suggest potential benefits in reducing diabetes, aiding dietary lipid digestion and absorption, and alleviating inflammation.

### 3.4. Phytochemical Characterization and Evaluation of the Antioxidant Potential of Supplemented Nougat

The phytochemical profile of the supplemented nougat formulas obtained by incorporating increasing concentrations (2, 4, and 6%) of beetroot skin powder is presented in Table 3. The findings emphasize that the enhanced nougat formulations, incorporating beetroot peel powder, increase betalains and polyphenols, creating an antioxidant-rich product.

Table 3 also indicates the bioactive compound stability of enriched nougat samples after 21 days of storage. The results indicate that during the 21 days of storage, the content of bioactive compounds in the supplemented nougat shows a slight decrease and, implicitly, a slightly lower antioxidant potential. However, nougat formulas supplemented with increasing concentrations of beetroot skin powder show a rich profile of betalains and polyphenols compared to the conventional product.

Regarding the antioxidant potential, the formulas of the nougat with added powder (4 and 6%) are superior to that of the control sample. Therefore, supplementing the nougat with concentrations of more than 2% beetroot skin powder contributes to its enrichment with bioactive compounds that lead to obtaining a product with high antioxidant activity.

Our results corroborate those of other studies. Alshehry [38] found that adding beetroot powder (7.5 or 10%) to muffins boosted the enriched items’ antioxidant, coloring, and antibacterial properties. Moreover, Amnah [48] investigated the impact of including beetroot powder (5 g) on the composition of biscuits. The findings indicated an enhancement in nutritional quality. The findings align with the research of Dhadage et al. [49] on multi-cereal snacks fortified with beetroot powder and Mitrevski et al. [50] on biscuits with 15%, 20%, and 25% beetroot powder.

### 3.5. Physicochemical Characterization

The enhanced nougat samples were examined from a physicochemical perspective, with the results shown in Table 4. The composition of nougat samples with beetroot powder differed significantly (*p* < 0.05).

The humidity content of the control sample was 3.51 ± 0.18 g/100 g, and with the rise in beetroot powder incorporation, the moisture content increased slightly; specifically, at the 6% level, the H3 nougat exhibited a moisture content of 3.69 ± 0.28 g/100 g. In all examined samples, a low humidity content was achieved, and the reduced moisture content combined with high production yield resulted in samples with a prolonged shelf life [51]. The control sample exhibited the lowest ash concentration at 0.42 ± 0.09 g/100 g, whereas the greatest ash content was recorded in nougat with 6% beetroot powder incorporation at 1.92 ± 0.18 g/100 g. The lipid concentration dropped with the augmentation of beetroot powder, resulting in H3 containing 2.91 ± 0.12 g/100 g.

The incorporation of beetroot powder results in a minor reduction in protein content. The concentration of insoluble fibers rose with the increasing proportion of beetroot powder, reaching 1.64 ± 0.21 g/100 g in the product containing 6% beetroot powder. Additionally, beetroot powder improved the sweets’ fiber content and water retention. The carbohydrate amounts decreased with beetroot powder addition and were the highest in the control sample, measuring 88.06 ± 0.25 g/100 g. Also, the energy value of the samples decreased in the samples with beetroot powder, but it was close to the conventional product. Beetroot powder added to cookies at 5–20% increased the protein from 7.39% to 9.12%, crude fiber from 0.95% to 1.90%, and ash from 0.93% to 1.89% [52].

Mitrevski et al. [50] produced biscuits with 15%, 20%, and 25% beetroot powder. Fresh biscuits made had 6.1–7.6% dietary fiber, 9.2–8.9% protein, and 30.6–35.9% sugar. Dietary fiber is acknowledged as a highly beneficial component in food products due to its ability to mitigate the risk of diabetes [53].

### 3.6. Analysis of the CIELAB Colorimetric Parameters of the Supplemented Nougat

Color is a crucial determinant of consumer approval for a product. The CIELAB colorimetric parameters analysis results of the nougat formulations enriched with varying concentrations of beetroot skin powder were expressed as L*, a*, and b* values (Table 5).

The luminosity of the beetroot powder samples markedly decreased, whereas the a* and b* values rose simultaneously with the addition of the powder compared to the control. As product dietary fiber increased, Uthumporn et al. [54] found that the L* coordinate value decreased.

**Figure 2:**
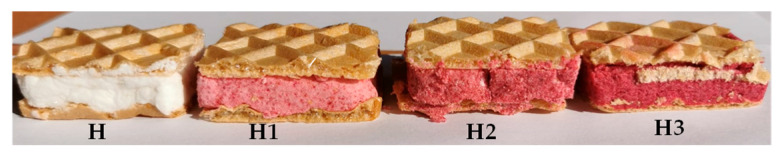
Nougat supplemented with beetroot skin powder: H—nougat without added beetroot skin powder; H1, H2, and H3—nougat formulas with the addition of 2, 4, and 6% (*w/w*) beetroot skin powder.

The results presented in Table 5 highlight the fact that by incorporating the beetroot skin powder in the composition of the nougat, it is characterized by red–violet shades (a* values), the intensity of the color being directly proportional to the percentage of powder added (2, 4, and 6%). The beetroot skin powder’s high coloring power and potential as a natural colorant in the nougat composition are confirmed by this consideration, which also enhances the product’s appeal to consumers (Figure 2).

The addition of beetroot powder in cookies reduced lightness (L*) and yellowness (b*) while enhancing redness (a*) [52]. Cookies with beet flour (10%, 15%, and 20%) exhibited higher a* values compared to b*, which is likely attributed to the elevated betalaine pigment content, especially betacyanins, which contribute to the reddish hue [55].

The Chroma, representing color intensity, had a trend similar to the parameter a*, which was highest in H3 and lowest in the control. The control sample exhibited a maximal hue angle value, while the sample that contained 2% beetroot skin powder had a minimal hue angle value. Beetroot skin powder supplementation enhanced ΔE, resulting in a 42.11 to 73.97 range in overall color change.

The nougat obtained had a moderate consistency, a purplish-red color specific to beetroot, a sweet and enjoyable taste, and a homogeneous texture specific to the conventional product.

### 3.7. Texture Analysis for Supplemented Nougat Samples

The texture of the nougat was analyzed instrumentally using the Textural Profile Analysis method. This method consists of a double penetration, which simulates mastication. The findings of the instrumental texture analysis are displayed in Table 6. The addition of beetroot skin powder increased the firmness and adhesion of the nougat samples. If the differences between the control sample and the sample with 2% added powder are not significant, when the amount of added powder increases, the differences become more obvious, with the sample with 6% added registering a firmness value almost three times higher than the control sample.

The evolution of firmness is due to the increase in the density and consistency of the paste when the powder is added. Firmness and adhesion positively influence product shape retention during storage. At the same time, the particles in the powder lead to the fragmentation of the protein matrix and the weakening of the internal bonds, a fact demonstrated by the decrease in cohesiveness with the increase in the amount of added powder. This makes the samples more easily disintegrate in the oral cavity during mastication.

Table 6 shows that the energy required to disintegrate the sample (chewability) decreases from 1.06 ± 0.06 mJ for the control sample to 0.41 ± 0.01 mJ for the sample with the most powder added. The beetroot skin powder added to the nougat also influences the elasticity of the samples. If the control sample has the capacity to recover 3.65 ± 0.005 mm of deformation, for the other samples, this capacity decreases proportionally with the powder percentage, reaching 6% to recover less than half (1.28 ± 0.01 mm). This behavior denotes the irreversible restructuring of the protein matrix during testing. In conclusion, it can be stated that the addition of beetroot skin powder has a positive effect on the texture of the nougat by improving firmness and facilitating mastication. Ingle et al. [52] improved the nutritional value of biscuits by incorporating varying concentrations of beetroot powder, specifically 0, 5, 7, 10, 15, and 20%. The hardness of the cookies increased with the augmentation of beetroot powder concentration. In a study by Holovko et al. [56], a sponge cake’s springiness and cohesiveness decreased, while its hardness increased due to the cake’s reduced hydration content. Sponge cakes with different amounts of beetroot powder (5, 10, 15, and 20% *w*/*w*) were obtained. Incorporating 15% beetroot powder into the sponge cake recipe markedly enhanced the structural parameters, with chewiness increasing by 2.9% compared to the control sample recipe.

### 3.8. Mineral Content of Supplemented Nougat Samples

Table 7 presents the mineral content analysis for the supplemented nougat samples without beetroot skin and those supplemented with beetroot skin at three concentrations (2%, 4%, and 6%).

The nougat samples showed significant differences in amounts of K, Mg, Na, P, Ca, Fe, Mn, Cu, and Zn (*p* < 0.05).

The calcium content increased significantly from 5.16 ± 0.07 mg/100 g in the control (H) to 6.65 ± 0.28 mg/100 g in the 6% supplemented sample (H3). This suggests that beetroot skin is a substantial source of calcium, and its inclusion markedly improved the mineral profile (*p* < 0.05). Phosphorus content rose from 13.61 ± 0.15 mg/100 g in H to 18.36 ± 0.35 mg/100 g in H3, showing a consistent and significant increase. Potassium, being one of the most abundant minerals in beetroot skin (Table 1), also showed a marked rise with supplementation—from 130.39 ± 1.03 mg/100 g (H) to 149.23 ± 1.12 mg/100 g (H3). This increase is significant and indicates potassium enrichment through supplementation.

Magnesium levels also increased from 8.11 ± 0.06 mg/100 g to 10.09 ± 0.20 mg/100 g, reflecting the beneficial mineral composition of beetroot skin and its contribution to micronutrient enhancement [57]. Although the values were small, the manganese content slightly increased from 0.01 ± 0.01 mg/100 g (H) to 0.02 ± 0.05 mg/100 g (H3). The increment was not statistically significant in all cases but suggests a trend toward improved trace mineral content. The same trend was for the copper content, which remained low but increased slightly from 0.01 ± 0.01 mg/100 g to 0.03 ± 0.03 mg/100 g, especially in the 6% sample. Iron content significantly increased from 0.04 ± 0.02 mg/100 g to 0.09 ± 0.10 mg/100 g. This doubling in value in the highest supplemented sample underlines the role of beetroot skin in iron enhancement. Sodium levels showed slight variations but no clear increasing trend (H: 125.31 ± 0.94, H3: 124.98 ± 0.91 mg/100 g), suggesting that beetroot skin powder did not significantly affect sodium content in the formulation. Zinc content increased from 0.02 ± 0.02 mg/100 g in the control to 0.05 ± 0.05 mg/100 g in the H3 sample, indicating a moderate but not statistically significant enrichment.

Table 1 illustrates that freeze-dried beetroot skin powder is abundant in potassium, sodium, phosphorus, magnesium, and calcium. Thus, beetroot skin powder is suitable for incorporation as a bioactive component of food items.

The nutritional value of supplemented hard candies increased significantly (*p* < 0.05) from 2% to 10% beetroot powder. Notable improvements were observed in dietary fiber (0.4–2.1 g/100 g, a 381% increase), potassium (0.6–2.8 mg/100 g, up by 405%), sodium (0.2–0.7 mg/100 g, a 331% rise), magnesium (0.05–0.26 mg/100 g, a 420% increase), and calcium (0.06–0.24 mg/100 g, showing a 300% improvement) [58]. Kaur et al. [59] reported increased levels of potassium (6.1 mg/100 g), sodium (1.53 mg/100 g), calcium (0.25 mg/100 g), and iron (0.01 mg/100 g) in supplemented candies formulated with 2% to 10% beetroot powder. Beetroot powder added to sponge cake up to 20% enhanced the mineral and fiber content [56].

### 3.9. Sensory Evaluation of Supplemented Nougat Samples

**Figure 3:**
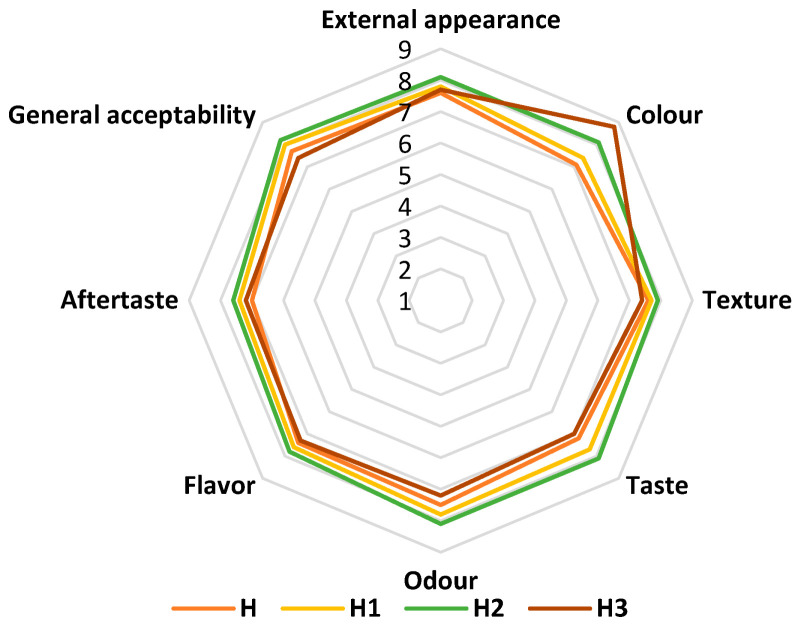
Comparative diagram of the specific sensory attributes of the supplemented nougat samples: H—nougat without the addition of beetroot skin powder; H1, H2, and H3—supplemented nougat with the addition of 2, 4, and 6% beetroot skin powder.

According to the sensory analysis of the supplemented nougat samples (Figure 3), the variants with beetroot skin powder had a balanced, pleasant color similar to beetroot, while the control variant was the least appreciated.

A positive assessment was received from tasters, who rated the supplemented nougat samples as having a slightly perceptible beetroot taste and aroma.

**Figure 4:**
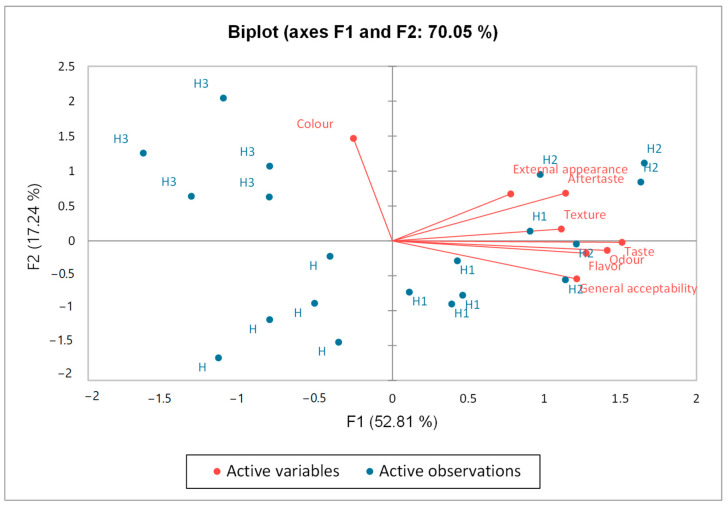
Illustration of relationships among sensory qualities utilizing Principal Component Analysis (PCA).

The biplot derived from PCA analysis (Figure 4) concisely represents the differences and correlations among the assessed sensory attributes and the batches of nougat supplemented with beetroot skin powder. The two axes account for 70.05% of the overall variation. Analyzing the PCA results, we can observe that sample H2 (the batch with 4% beetroot powder) emerged as the frontrunner regarding consumer preferences. The external appearance, color, aftertaste, and texture characteristics, located in the upper-right quadrant, were perceived by consumers as positive. Taste, odor, flavor, and general acceptability also positively contribute to axis F1, as with the color attribute.

The most popular nougat was H2, with 4% powder. As beetroot skin powder percentage increased, tasters disliked the texture and aftertaste. In a study by Lucky et al. [12], the nutritional and sensory research showed that the cake with 15% (*w*/*w*) beetroot powder was more pleasurable than the others (0, 5, 10, and 20%).

Ingle et al. [52] conducted a study to enhance the nutritious properties of cookies by including varying doses of beetroot powder, specifically 0, 5, 7, 10, 15, and 20%. The sensory examination of cookies determined that those made with 10% beetroot powder were more pleasant than the rest. Healthy, natural products have led to the development of innovative products with increased sensory characteristics (taste, scent, color, and texture) to meet consumer demand [60]. Thus, the obtaining of a new variety of nougat, with the addition of beetroot skin powder, is distinguished by a red–violet color, conferred by the addition of powder rich in pigments (betalains) from beetroot, attractive to consumers, especially children. The product’s added value is highlighted by the high intake of natural antioxidants present in beetroot skins, which have remarkable antioxidant potential and are free of toxicity. In addition, substituting chemically synthesized additives with natural ones present in beetroot skin brings many benefits and directly contributes to increasing the quality of life.

## 4. Conclusions

This study provides a novel way to use beetroot by-products as bioactive ingredients for value-added goods. The beetroot skin powder extract had high polyphenolic content and antioxidant activity. Beetroot skin powder extract showed in vitro inhibitory efficacy against α-amylase, α-glucosidase, lipase, and lipoxygenase, suggesting it may help manage metabolic syndrome-related enzymes.

The obtained results demonstrate the multifunctionality of the powder obtained from the beetroot skins in the composition of the nougat as an important source of natural compounds with antioxidant, coloring, and flavoring activity, which improve the nutritional and sensory characteristics, such as the color and aroma of the final product. Also, the addition of beetroot skin powder has a positive effect on the texture of the supplemented nougat by improving firmness and facilitating chewing.

This study offers insightful information about the potential use of beetroot skin powder as a bioactive powder in nougat, improving the confection’s nutritional value and sensory appeal while providing a sustainable solution to food waste. The results add to the broader discourse on novel methods for producing value-added food items, encouraging customers to make sustainable and health-conscious decisions.

## Figures and Tables

**Table 1:** Global phytochemical characterization and color parameters of beetroot skin powder extract.

Parameter		Beetroot Skin Powder Extract
Total betalains, mg/g dw		1.24 ± 0.04
Total flavonoids, mg CE/g dw		104.12 ± 0.84
Total polyphenols, mg GAE/g dw		212.14 ± 1.49
Antioxidant activity, µM Trolox/g dw	DPPH	33.42 ± 1.12
	ABTS	46.69 ± 0.31
Inhibition, %		93.32 ± 0.68
L*		36.90 ± 0.43
a*		30.99 ± 0.41
b*		5.11 ± 0.06
Moisture, %		8.05 ± 0.71
Ash, %		6.51 ± 0.48
Fat, %		0.59 ± 0.09
Protein, %		17.12 ± 0.74
Carbohydrates, % of which Total dietary fiber, %		67.73 ± 1.20 33.10 ± 1.22
Calcium (Ca, mg/100 g)		27.82 ± 0.81
Phosphorus (P, mg/100 g)		90.87 ± 1.02
Potassium (K, mg/100 g)		282.60 ± 1.12
Magnesium (Mg, mg/100 g)		26.31 ± 0.79
Manganese (Mn, mg/100 g)		0.46 ± 0.09
Iron (Fe, mg/100 g)		0.84 ± 0.52
Copper (Cu, mg/100 g)		0.06 ± 0.02
Sodium (Na, mg/100 g)		76.96 ± 0.99
Zinc (Zn, mg/100 g)		0.44 ± 0.22

**Table 2:** The beetroot skin powder extract’s α-amylase, α-glucosidase, lipase, and lipoxygenase inhibitory capacities (IC50 values; μg/mL).

Sample	IC50 (μg/mL)			
	α-Amylase	α-Glucosidase	Lipase	Lipoxygenase
Extract	4.22 ± 0.40 ^a^	3.24 ± 0.27 ^a^	1.05 ± 0.23 ^b^	5.24 ± 0.59 ^a^
Acarbose	2.69 ± 0.08 ^b^	1.78 ± 0.06 ^b^	-	-
Orlistat	-	-	3.35 ± 0.24 ^a^	-
Quercetin	-	-	-	2.40 ± 0.10 ^b^

Values in a column that do not share the same letter differ significantly (*p* < 0.05).

**Table 3:** Phytochemical characteristics of the supplemented nougat and stability during 21 days of storage: H—H-nougat without the addition of beetroot skin powder; H1, H2, and H3—nougat formulas with the addition of 2, 4, and 6% (*w*/*w*) beetroot skin powder.

Sample	Phytochemical		0 Days	7 Days	14 Days	21 Days
H	Total betalain content (mg/100 g dw)		Nd *	Nd *	Nd *	Nd *
	Total polyphenol content (mg GAE/100 g dw)		32.95 ± 4.19 ^a^	30.00 ± 1.99 ^a^	26.76 ± 1.82 ^ab^	21.71 ± 1.20 ^b^
	Antioxidant activity (µM Trolox/100 g dw)	DPPH	2.68 ± 0.36 ^a^	2.67 ± 0.09 ^a^	2.28 ± 0.02 ^ab^	2.02 ± 0.05 ^b^
		ABTS	3.86 ± 0.22 ^a^	3.12 ± 0.14 ^a^	3.10 ± 0.85 ^a^	2.99 ± 0.21 ^ab^
H1	Total betalain content (mg/100 g dw)		1.78 ± 0.08 ^a^	1.49 ± 0.03 ^b^	1.17 ± 0.06 ^c^	1.02 ± 0.09 ^c^
	Total polyphenol content (mg GAE/100 g dw)		38.63 ± 1.26 ^a^	33.36 ± 1.81 ^b^	30.73 ±0.93 ^b^	25.46 ± 0.90 ^c^
	Antioxidant activity (µM Trolox/100 g dw)	DPPH	25.20 ± 0.81 ^a^	24.51 ± 0.78 ^a^	19.40 ±1.02 ^b^	16.21 ± 0.25 ^c^
		ABTS	37.21 ± 0.36 ^a^	36.50 ± 1.02 ^a^	34.89 ± 1.01 ^b^	33.74 ± 0.63 ^b^
H2	Total betalain content (mg/100 g dw)		2.86 ± 0.03 ^a^	2.56 ± 0.04 ^b^	2.19 ± 0.16 ^c^	1.99 ± 0.06 ^c^
	Total polyphenol content (mg GAE/100 g dw)		53.44 ± 1.33 ^a^	50.87 ± 0.62 ^a^	46.45± 1.67 ^b^	40.48 ± 0.76 ^c^
	Antioxidant activity (µM Trolox/100 g dw)	DPPH	54.94 ± 2.67 ^a^	50.55 ± 0.71 ^b^	46.42 ± 0.97 ^c^	41.47 ± 1.17 ^d^
		ABTS	62.12 ± 1.2 ^a^	60.23 ± 0.52 ^a^	59.14 ± 0.63 ^b^	58.21 ± 1.14 ^b^
H3	Total betalain content (mg/100 g dw)		3.77 ± 0.09 ^a^	3.52 ± 0.09 ^b^	3.25 ± 0.11 ^c^	2.83 ± 0.08 ^d^
	Total polyphenol content (mg GAE/100 g dw)		69.48 ± 2.88 ^a^	66.42 ± 1.82 ^ab^	61.55 ± 1.34 ^b^	53.65 ± 0.89 ^c^
	Antioxidant activity (µM Trolox/100 g dw)	DPPH	73.89 ± 3.65 ^a^	66.86 ± 1.59 ^b^	59.91 ± 2.37 ^c^	53.33 ± 1.92 ^d^
		ABTS	81.06 ± 1.14 ^a^	78.14 ±0.63 ^a^	74.96 ± 0.85 ^b^	72.94 ± 1.20 ^c^

* Nd: undetectable. Different letters (a–d) for the same parameter (per line) show significant differences between means (*p* < 0.05).

**Table 4:** Physicochemical characteristics of the supplemented nougat samples.

Physical-Chemical Characteristics	H	H1	H2	H3
Proteins, g/100 g	4.61 ± 0.12 ^a^	4.34 ± 0.11 ^b^	4.11 ± 0.07 ^c^	4.01 ± 0.09 ^c^
Lipids, g/100 g	3.40 ± 0.17 ^a^	3.26 ± 0.15 ^b^	3.03 ± 0.14 ^c^	2.91 ± 0.12 ^d^
Carbohydrates, g/100 g	88.06 ± 0.25 ^a^	87.89 ± 0.33 ^b^	87.80 ± 0.27 ^b^	87.47 ± 0.30 ^c^
Insoluble fibers, g/100 g	0.00 ± 0.00 ^c^	1.10 ± 0.10 ^b^	1.76 ± 0.13 ^a^	1.64 ± 0.21 ^a^
Humidity, g/100 g	3.51 ± 0.18 ^b^	3.55 ± 0.19 ^ab^	3.61 ± 0.24 ^a^	3.69 ± 0.28 ^a^
Ash, g/100 g	0.42 ± 0.09 ^d^	0.96 ± 0.12 ^c^	1.45 ± 0.15 ^b^	1.92 ± 0.18 ^a^
Energetic value, %: Kcal/100 g kJ/100 g	401.28 ± 0.25 ^a^ 1677.35 ± 0.25 ^a^	400.46 ± 0.22 ^a^ 1673.92 ± 0.22 ^a^	398.43 ± 0.19 ^b^ 1665.44 ± 0.19 ^b^	395.39 ± 0.17 ^c^ 1652.73 ± 0.17 ^c^

Different letters (a–d) for the same parameter (per line) show significant differences between means (*p* < 0.05).

**Table 5:** Colorimetric parameters of the nougat samples: H—nougat without the addition of beetroot skin powder; H1, H2, and H3—nougat formulas with the addition of 2; 4 and 6% (*w*/*w*) beet skin powder.

Sample	L*	a*	b*	Chroma	Hue Angle	ΔE
H	104.79± 0.45 ^a^	7.08± 0.06 ^c^	5.57 ± 0.48 ^b^	9.01± 0.23 ^c^	0.67± 0.09 ^a^	-
H1	75.06 ± 0.35 ^b^	36.90 ± 2.59 ^b^	5.51 ± 1.16 ^b^	37.31± 0.65 ^b^	0.15± 0.03 ^b^	42.11± 0.23 ^c^
H2	53.11 ± 0.50 ^c^	42.50 ± 1.33 ^ab^	6.38± 0.90 ^ab^	42.98± 0.23 ^a^	0.16± 0.05 ^b^	62.66± 0.23 ^b^
H3	41.47 ± 1.10 ^d^	45.22 ± 3.57 ^a^	8.24 ± 1.03 ^a^	45.96± 0.23 ^a^	0.18± 0.06 ^b^	73.97± 0.23 ^a^

Different letters (a–d) in the column for the same analyzed parameter show significant differences between means (*p* < 0.05).

**Table 6:** Textural parameters of the nougat samples: H—nougat without the addition of beetroot skin powder; H1, H2, and H3—nougat with the addition of 2, 4, and 6% (*w/w*) beetroot skin powder.

Parameter	H	H1 (2%)	H2 (4%)	H3 (6%)
Firmness, N	0.54 ± 0.05 ^a^	0.56 ± 0.01 ^a^	1.06 ± 0.12 ^b^	1.51 ± 0.03 ^c^
Adhesion, mJ	1.00 ± 0.03 ^a^	1.11 ± 0.18 ^a^	1.42 ± 0.02 ^a^	1.55 ± 0.03 ^a^
Cohesiveness	0.57 ± 0.03 ^a^	0.45 ± 0.01 ^a^	0.43 ± 0.01 ^a^	0.38 ± 0.005 ^a^
Elasticity, mm	3.65 ± 0.005 ^a^	3.23 ± 0.05 ^a^	2.32 ± 0.15 ^a^	1.28 ± 0.01 ^a^
Gumminess, N	0.29 ± 0.005 ^a^	0.26 ± 0.04 ^a^	0.23 ± 0.02 ^a^	0.17 ± 0.05 ^a^
Chewability, mJ	1.06 ± 0.06 ^a^	0.70 ± 0.02 ^ab^	0.52 ± 0.01 ^ab^	0.41 ± 0.01 ^c^

Differences between the analyzed samples are highlighted by lowercase letters per row. Mean values that share a letter are not significantly different (*p* > 0.05).

**Table 7:** Mineral composition of nougat samples supplemented with beetroot skin powder.

Parameters	H	H1 (2%)	H2 (4%)	H3(6%)
Calcium (Ca, mg/100 g)	5.16 ± 0.07 ^d^	5.47 ± 0.11 ^c^	5.99 ± 0.15 ^b^	6.65 ± 0.28 ^a^
Phosphorus (P, mg/100 g)	13.61 ± 0.15 ^c^	13.90 ± 0.19 ^c^	15.54 ± 0.26 ^b^	18.36 ± 0.35 ^a^
Potassium (K, mg/100 g)	130.39 ± 1.03 ^d^	136.02 ± 0.89 ^c^	140.92 ± 0.95 ^b^	149.23 ± 1.12 ^a^
Magnesium (Mg, mg/100 g)	8.11 ± 0.06 ^d^	8.97 ± 0.15 ^c^	9.68 ± 0.17 ^b^	10.09 ± 0.20 ^a^
Manganese (Mn, mg/100 g)	0.01 ± 0.01 ^a^	0.01 ± 0.02 ^a^	0.02 ± 0.04 ^a^	0.02 ± 0.05 ^a^
Iron (Fe, mg/100 g)	0.04 ± 0.02 ^b^	0.05 ± 0.14 ^ab^	0.07 ± 0.17 ^a^	0.09 ± 0.20 ^a^
Copper (Cu, mg/100 g)	0.01 ± 0.01 ^a^	0.01 ± 0.02 ^a^	0.02 ± 0.03 ^a^	0.03 ± 0.03 ^a^
Sodium (Na, mg/100 g)	125.31 ± 0.94 ^a^	122.38 ± 0.85 ^d^	123.97 ± 0.88 ^c^	124.98 ± 0.91 ^b^
Zinc (Zn, mg/100 g)	0.02 ± 0.02 ^a^	0.03 ± 0.02 ^a^	0.04 ± 0.04 ^a^	0.05 ± 0.05 ^a^

Superscripts with distinct letters in a row are statistically different (*p* < 0.05).

## Data Availability

The original contributions presented in this study are included in the article. Further inquiries can be directed to the corresponding authors.
